# Synthesis of FAU-Type Zeolite Membranes with Antimicrobial Activity

**DOI:** 10.3390/molecules25153414

**Published:** 2020-07-28

**Authors:** T. Jean Daou, Thomas Dos Santos, Habiba Nouali, Ludovic Josien, Laure Michelin, Laurent Pieuchot, Patrick Dutournie

**Affiliations:** 1 Institut de Science des Matériaux de Mulhouse (IS2M), Université de Haute Alsace (UHA), CNRS, UMR 7361, 3 bis rue Alfred Werner, F-68093 Mulhouse, France; thomas.dos-santos@uha.fr (T.D.S.); habiba.nouali@uha.fr (H.N.); ludovic.josien@uha.fr (L.J.); laure.michelin@uha.fr (L.M.); laurent.pieuchot@uha.fr (L.P.); patrick.dutournie@uha.fr (P.D.); 2 Université de Strasbourg (UniStra), F-67000 Strasbourg, France

**Keywords:** FAU-type zeolite, membrane, zeolites, in-situ synthesis, ionic exchange, bactericidal activity

## Abstract

In this study, a layer of a pure and dense phase of FAU-type zeolite was synthesized directly on the surface of α-Al_2_O_3_ plane macroporous support. Before hydrothermal synthesis, a step of cleaning of the support by an anionic detergent was performed, a roughness surface is created, allowing the anchoring of the zeolite nuclei and then their growth, favoring in this sense the formation of a homogeneous zeolite layer. The obtained membranes were fully characterized using X-ray diffraction analysis (XRD), nitrogen sorption, scanning electron microscopy (SEM), and mercury porosimetry. After 24 h of thermal treatment at 75 °C, a homogeneous zeolite layer composed of bipyramidal crystals of FAU-type zeolite is obtained with a thickness of about 2.5 µm. No obvious defects or cracks can be observed. It was found that the increase in heating temperature could lead to the appearance of an impurity phase, GIS-type zeolite. Then the ideal zeolite membrane was exchanged with Ag^+^ or Zn^2+^ cations to studies their antimicrobial properties. Zeolites membranes exchanged with Ag^+^ showed an agar-diffusive bactericidal activity against gram negative *Escherichia coli* (*E. coli*) bacteria. Zn^2+^ exchanged zeolite membrane presented a bacteriostatic activity that is less diffusive in agar. As expected, non-exchanged zeolite membrane (in its Na^+^ form) have no effect on bacterial activity. This process is particularly interesting for the synthesis of a good quality FAU-type zeolite membranes with antimicrobial properties.

## 1. Introduction

Zeolites are a microporous crystalline aluminosilicate with a three-dimensional network composed of SiO_4_ and AlO_4_ tetrahedrally interconnected by oxygen bridges [1,2]. Due to their excellent textural properties and high thermal (up to 700 °C) and chemical stabilities, zeolite materials offer unique frames for a wide variety of industrial applications (catalysis, molecular decontamination, separation, adsorption, energy storage, etc.). The substitution of Si atoms by Al atoms in the zeolite framework creates negative charge that should be compensated by cations (the most common compensating cations are Na^+^). These cations can be easily exchanged by silver, copper, or zinc cations to confer to zeolites antibacterial, antiviral, and antifungal activities [3,4,5,6,7,8,9]. Silver-exchanged zeolites have antibacterial and antifungal properties against a broad spectrum of microscopic bacteria and fungi (*Escherichia Coli*, *Pseudomonas aeruginosa*, *Bacilus cereus*, *Staphylococcus aureus*, *Candida albicans*, *Candida glabata*, *Aspergillus niger* and *Penicillium vinaceum*, etc.). They can be considered as a credible alternative to materials functionalized with silver nanoparticles, because their activity is similar or even higher. Generally, the antibacterial activity depends on the silver content, but certain zeolites exchanged with silver show a significant efficiency in the elimination of bacteria, despite their very low silver content (<0.2%) [5]. In the case of zeolites, not only the silver content, but other parameters (structural type, crystal size, and Si/Al ratio of the framework) can influence the activity [3,4,5,6,7,8,9].

Zeolites exchanged with silver face a great problem which is their high cost. It is known that other transition metals such as copper, zinc, etc. can show antibacterial properties at a much lower cost [9]. Thus, zeolites exchanged by these metal cations have been prepared and tested for their antibacterial properties. Milenkovic et al. [9], studied the antibacterial activity of zeolites exchanged with copper and zinc towards *E. coli* and compared with that of zeolites exchanged with silver. A natural clinoptilolite zeolite (NZ) and a zeolite A (LTA, Linde 4A) were exchanged with transition metal ions. All materials showed a similar content of transition metals—0.24–0.28 mmol/g of zeolite. The exchange of copper and zinc has been shown to lead to a sharp increase in antibacterial activity compared to non-exchanged zeolites with the exception of the Zn-NZ zeolite. However, the effectiveness of these materials remains lower than that of zeolites exchanged with silver. Antibacterial activity decreases as follows: Ag-NZ ≈ Ag-A > Cu-NZ ≈ Cu-A > Zn-NZ >> Zn-A. The release of zinc and copper ions into the solution was considerably less than that of silver, suggesting that the mechanism of antibacterial activity in exchanged zeolites is directly related to the materials and not to the released ions. In general, the bacteria removal efficiency of the zeolite varies depending on the nature of the metal cation used in the following order: Ag^+^ > Cu^2+^ > Fe^3+^ > Zn^2+^ > Ni^2+^.

Unfortunately, conventional syntheses of zeolites give often rise to powders composed of micron-size crystals. These powders cannot be used directly for industrial applications, especially for water purification and molecular sieving [10]. A shaping step has to occur before using them. Zeolite membranes are potentially used in industry for molecular separation, water treatment, electrical sensors and insulation because of their high thermal stability and uniform pore size [10,11,12,13,14,15]. Various types of zeolitic membranes, especially MFI [16,17], SOD [18], LTA [10,18] and FAU [19,20], have been reported for molecules or gaseous separation and/or adsorption. In particular, FAU-type zeolite membranes are suitable for the separation of large molecules due to their 12-membered oxygen rings pore openings with a diameter of 0.74 nm [21]. These zeolite membranes can be synthesized by direct in situ crystallization or by seeding and secondary growth process [22,23,24,25,26,27]. However, it appears that the direct in situ synthesis of FAU-type zeolite membranes without defect remains a challenge because of the low heterogeneous nucleation of the zeolite on the used support. Mainly intended to cover the surface of the support with a layer of zeolite seeds before the growth step, the secondary growth process leads to the formation of a uniform and dense layer on the surface of the support. Compared with in-situ hydrothermal growth method, the secondary growth method has more advantages for manipulating the microstructure of the membrane, in particular for adjusting the thickness and orientation of the crystals, since the nucleation process can be separated from crystalline growth [23].

To date, various techniques have been developed to improve the direct in-situ crystallization of homogeneous FAU-type zeolite membrane on macroporous alumina support. Huang, et al. [28,29] and Zhou, et al. [30] used 3-aminopropyltriethoxysilane (APTES) as covalent linker between the FAU-type zeolite layer and the porous alumina support. The 3-aminopropylsilane moieties have been used as highly effective molecular binders to promote the binding and anchoring of zeolite nuclei to the macroporous alumina surface during hydrothermal synthesis. Zhou, et al. [31] used polydopamine (PDA) to prepare dense pure phase FAU-type zeolite membranes on the surface of PDA-modified α-Al_2_O_3_ macroporous tubes. It was found that PDA facilitated the growth of the FAU-type zeolite layer on the α-Al_2_O_3_ tubes and reduced the synthesis time to obtain a high quality FAU-type zeolite membrane.

In this project, a strategy was developed to synthesize highly crystallized FAU-type zeolite membranes by in situ crystallization and in the absence of organic structuring. The zeolite layer was generated directly on the surface of the macroporous α-Al_2_O_3_ plates treated with Alconox^®^ an anionic detergent, without the use of any other binder or tie layer. The ideal FAU-type zeolite membranes were then exchanged with silver and zinc cations to confer them antibacterial properties.

## 2. Results and Discussion

### 2.1. Hydrothermal Synthesis of FAU-Type Zeolite Membranes

**Figure 1:**
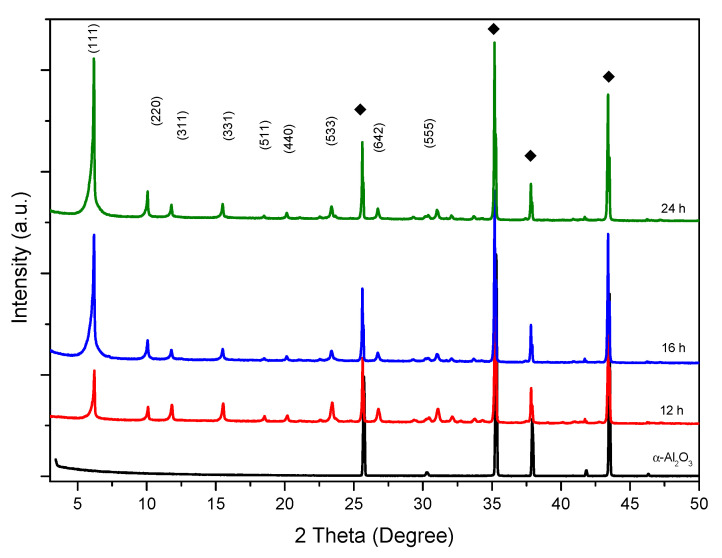
X-ray diffraction (XRD) patterns of FAU-type zeolite membranes prepared on Alconox^®^-treated α-Al_2_O_3_ supports at 75 °C for 12, 16, and 24 h. (Black diamonds indicate the peaks of α-Al_2_O_3_ support).

The treated supports were immersed in the synthesis solution as described in Section 3.3 and subjected to hydrothermal treatment. The influence of the synthesis time on the growth of the film has been studied. The synthesis time varied from 12 to 24 h. Zeolitic membrane growth and their crystallization state were investigated using X-ray diffraction (XRD) technique, which shows characteristic patterns of pure FAU-type zeolite in agreement with the corresponding pattern available (Pattern 01-070-4281 from International Center of Diffraction Data (ICDD)) [32,33,34] (Figure 1) with additional peaks at high 2θ angles attributed to α-Al_2_O_3_ composing the support [24,35]. The crystallinity of the synthesized FAU-type zeolite membranes increases progressively with the crystallization time as shown by the increase of the intensity of the XRD diffraction peaks in Figure 1. XRD patterns were indexed in the cubic symmetry (space group Fd-3m) and lattice parameters (a_0_) were determined with STOE Win X Pow software [36]. From these parameters, the Si/Al framework ratios were deduced using Breck and Flanigen’s equation:

(Si/Al = ((192 × 0.00868)/(a_0_ − 24.191)) − 1), (1)

with a_0_ cell parameter [32,37]. A silicon to aluminum molar ratio around 1.2 is obtained for the synthesized FAU-type zeolite membranes. This Si/Al ratio of 1.2 is attributed to zeolite X of FAU-type [21,32].

**Figure 2:**
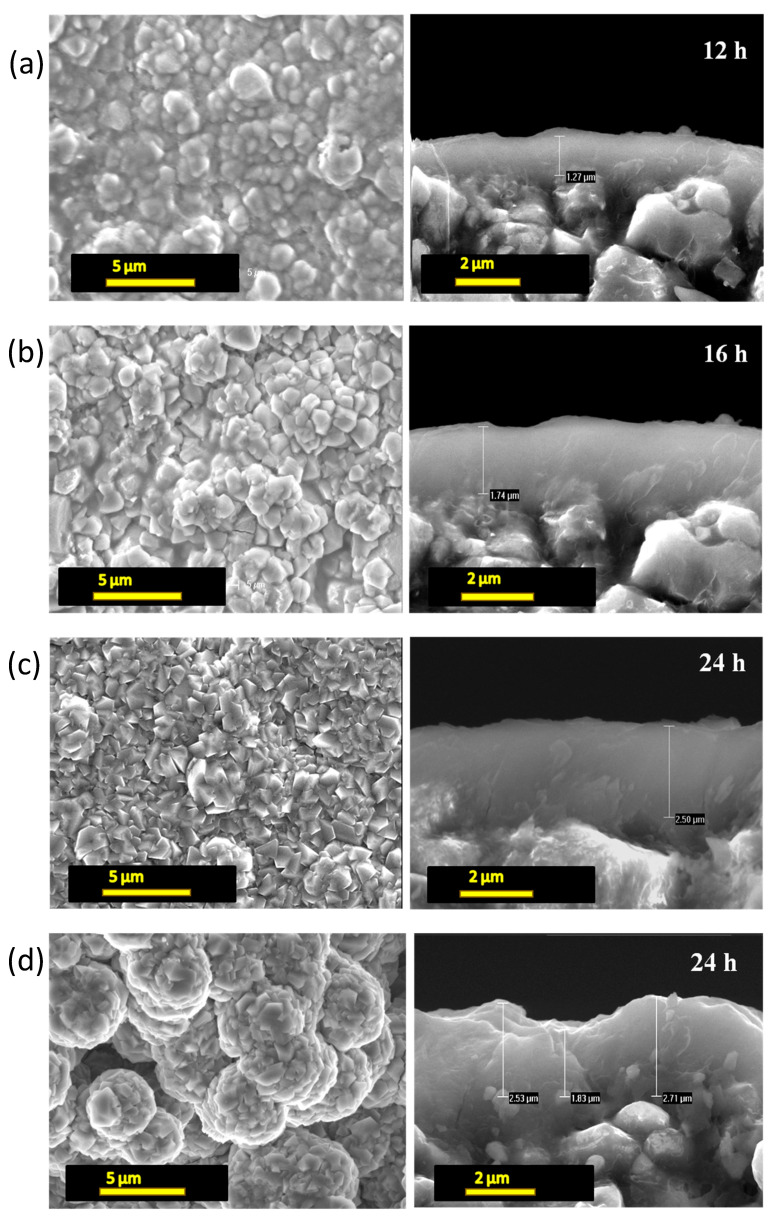
Scanning electron microscopy (SEM) images of FAU-type zeolite membranes prepared on Alconox^®^-treated (**a**–**c**) α-Al_2_O_3_ support at 75 °C for 12, 16 and 24 h. SEM images of FAU-type zeolite membranes prepared on untreated (**d**) α-Al_2_O_3_ support at 75 °C for 24 h. Top view (left); cross-section of the surface (right).

Scanning electron microscopy (SEM) investigations were performed on the obtained FAU-type zeolite membranes (Figure 2). As shown in Figure 2a, after 12 h of synthesis, crystals are observed and a thin layer of a thickness of about 1 micron covers the entire surface of the support. After 16 h of treatment (Figure 2b) the bipyramidal morphology of FAU-type zeolite crystals was observed confirming the increase of crystallinity as observed above from XRD patterns. At this point, a continuous layer of FAU-type zeolite with a thickness of about 1.7 µm is formed on the surface of the support. The crystals continue to grow until reaching their final bypyramidal morphology after 24 h of thermal treatment. As shown in Figure 2c, after 24 h of synthesis, the FAU-type zeolite crystals are well crystallized (in correlation with XRD results) and a layer of FAU-type zeolite of 2.5 μm in thickness is obtained. Thus, under these ideal conditions of in situ hydrothermal synthesis in one step, a dense, crack-free, FAU-type zeolite membrane is formed on the treated α-Al_2_O_3_ macroporous support.

In order to show the importance of the Alconox^®^ treatment step, the same synthesis was done at 75 °C for 24 h using this time an untreated α-Al_2_O_3_ macroporous support. As it is shown in Figure 2d a set of non-homogeneous aggregates of FAU-type zeolite crystals are formed on the surface of the α-Al_2_O_3_ support instead of a homogeneous FAU-type zeolite film once treated α-Al_2_O_3_ macroporous support is used. This result confirms the importance of Alconox^®^ treatment step on the formation of homogenous zeolite layer as already observed in our previous paper [24,35,38,39,40].

**Figure 3:**
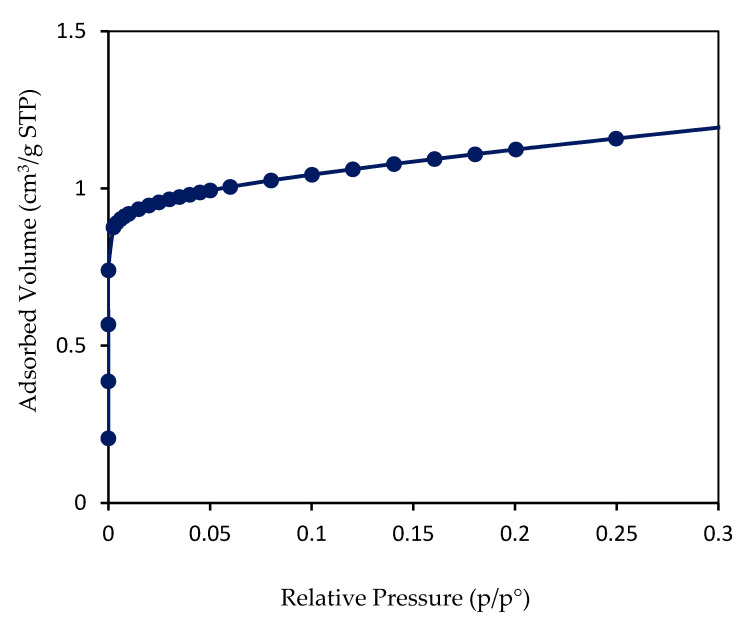
Nitrogen adsorption isotherm at −196 °C of the FAU-type membrane after 24 h of thermal treatment. The adsorbed volume is expressed in cm^3^ per gram of the total sample, including the mass of the alumina support.

The nitrogen sorption properties of the best FAU membrane obtained after Alconox^®^ treatment of the substrate and a thermal treatment at 75 °C for 24 h were investigated to evaluate the micropores accessibility and to determine the amount of zeolite deposited on the surface of α-Al_2_O_3_ support. Figure 3 reports the nitrogen adsorption isotherm of the obtained zeolite membrane. The textural properties are shown in Table 1. The adsorption isotherm is of type I at low relative pressure, according to the International Union of Pure and Applied Chemistry (IUPAC) classification [41,42] which is characteristic of microporous materials. It should be noted that the sample was degassed at 300 °C for 15 h under vacuum, without going through a calcination step due to the absence of organic template in the zeolite framework. This temperature was chosen to eliminate the physisorbed water and Volatile Organic Compounds (VOC) molecules. According to the nitrogen adsorption isotherm, the zeolite layer formed has a total adsorbed volume of 1.04 cm^3^/g at Standard conditions of Temperature and Pressure (STP) at relative pressure of 0.1, including the mass of the support (which does not adsorb nitrogen), the microporous volume of zeolite layer could be deduced: 1.6 × 10^−3^ cm^3^/g (see Table 1).

Knowing this value and comparing it with that obtained for the equivalent powder from the same composition of FAU-type zeolite synthesis (207 cm^3^/g STP), makes it possible to determine the mass of zeolite deposited on the support. Equation 2 is based on the assumption that all the porosity of the zeolite layer is accessible [38,39,40,43]. It is possible to calculate the mass of the zeolite layer [38,39,40,43]. The mass of the sample (m_total_ (sample)), including the support and the zeolite layer, is determined by weighing (m_total_ = 4.57 g) after degassing. On the basis of this equation, the α-Al_2_O_3_ support could be coated with 22.9 mg of FAU-type zeolite, [(1.04 cm^3^/g × 4.57 g)/207 cm^3^/g]. This result is in agreement with the mass composition determined by weighing. In consequence, the whole porosity of the microporous film is accessible.

(2) $$m\left(layer\right)=\frac{V\left(sample\right)\times m_{total}\left(sample\right)}{V\left(equivalent\;powder\right)}$$

**Figure 4:**
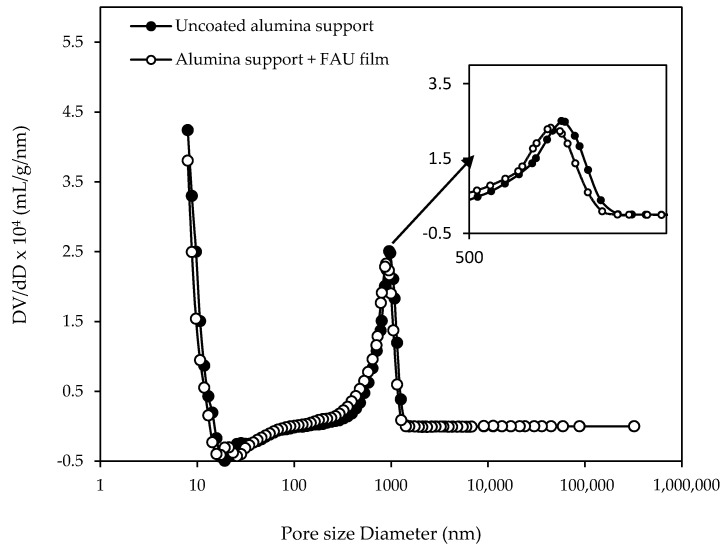
Pore size distribution of the uncoated alumina support (solid symbol) and the coated alumina support with FAU-type zeolite layer after 24 h of thermal treatment (empty symbol) performed by mercury porosimetry with a zoom on the region with pore size diameter between 500 and 1100 nm.

The mercury intrusion experiment carried out on the best FAU-type membrane (and the uncoated alumina substrate as reference) confirmed the formation of a continuous zeolite film on the surface of the alumina macroporous support (Figure 4). A reduction by approximately 8% of the mean macropore diameter from 967 nm (uncoated alumina substrate) to 886 nm (alumina substrate coated with zeolite layer) is observed on the pore size distributions shown in Figure 4. Macropores, therefore remain accessible.

### 2.2. Ion-Exchange

**Figure 5:**
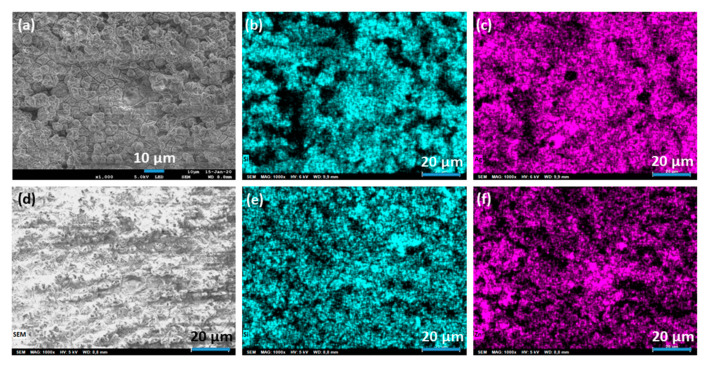
SEM images of FAU-zeolite membranes exchanged with silver (**a**) or zinc (**d**) and Energy Dispersive X-ray (EDX) mapping of silicon (**b**,**e**), silver (**c**) and zinc (**f**) elements present in these FAU-zeolite membranes.

**Figure 6:**
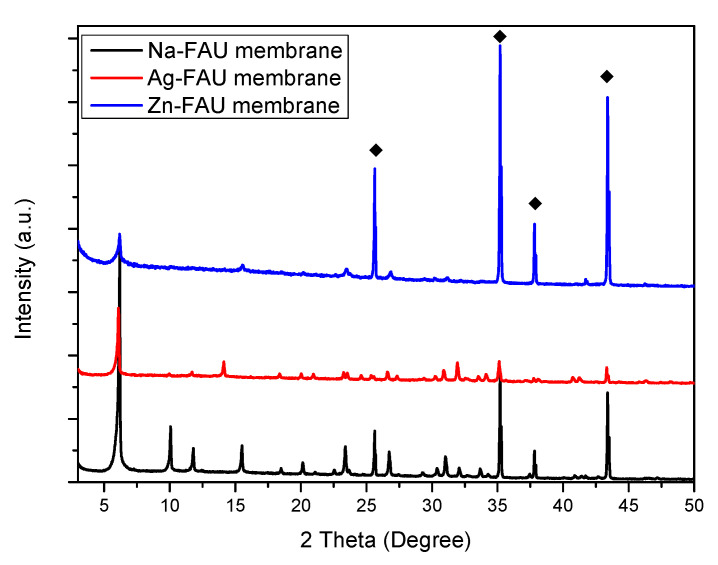
XRD patterns of Na-FAU membrane on α-Al_2_O_3_ supports, silver exchanged FAU membrane and zinc exchanged FAU membrane. Black diamonds indicate the peaks of α-Al_2_O_3_ support.

FAU-type zeolite membrane 2 × 2 cm^2^ obtained after Alconox^®^ treatment and a thermal treatment at 75 °C for 24 h were cut into different small membranes then ion exchange was done and repeated on three different small membranes for each cation. Scanning electron microscopy and Energy Dispersive X-ray (EDX) mapping of the FAU-type membrane obtained after ionic exchange with silver or zinc salts are shown in Figure 5. Inspection of these results reveals that the replacement of Na^+^ compensating cations (present in the microporosity of the zeolite to counter balance the negative charge generated by the presence of aluminum in the zeolite framework) by Ag+ and Zn^2+^ does not affect the morphology of FAU-type zeolites. For the silver-treated membranes and zinc-treated membranes a homogeneous distribution of silver and zinc is observed in Figure 5c,f. The EDX mapping of silicon in Figure 5b,e shows the presence at the surface of the exchanged membranes of a homogeneous layer of Si elements confirming that zeolites are still present at the surface. This result was then confirmed by XRD patterns shown in Figure 6 which show that all the identified peaks on exchanged zeolite membranes are attributed to FAU-type zeolite (Pattern 01-079-1884 for Ag exchanged FAU-type zeolite and pattern 04-009-7258 for Zn exchanged FAU-type zeolite, ICDD) and α-Al_2_O_3_. Changes in peaks intensities and slight shifts are observed when sodium cations are replaced by other silver or zinc cations. These results were already observed in the literature [33,44,45,46,47,48] and were explained as a consequence of the difference of the scattering power which is specific to each cation and also by a slightly different sites occupation in the pores [33,48,49].

### 2.3. Antibacterial Activity of the Zeolitic Membrane

The antibacterial activity of the zeolite samples was tested against gram-negative Escherichia coli (*E. coli*) bacteria via the agar diffusion method, colony-forming unit counting and live/dead staining analyzed by confocal microscopy.

#### 2.3.1. Inhibition through Diffusion

**Figure 7:**
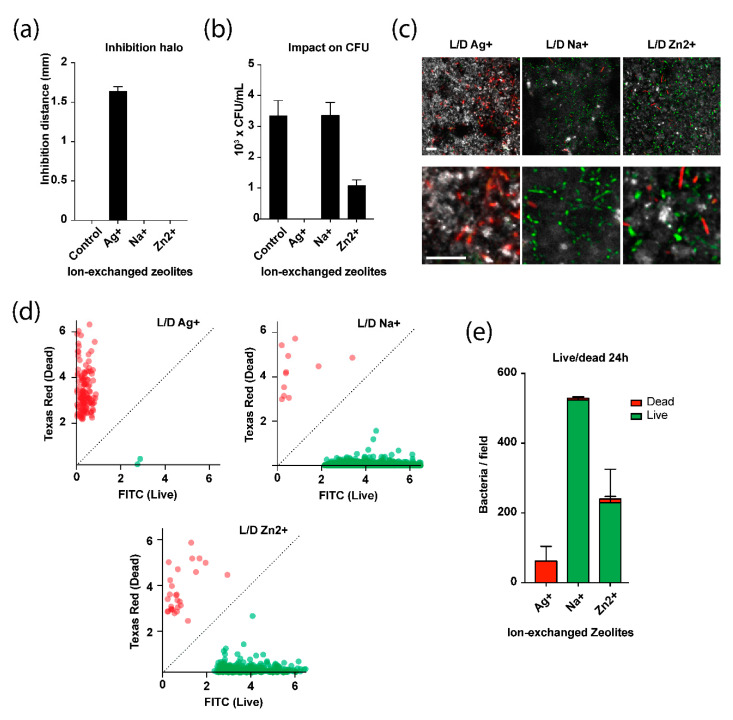
Antimicrobial activity of ion-exchanged zeolites. (**a**) Inhibition distances of *E. coli* bacteria growth by Na-FAU zeolite membrane and ion-exchanged (Ag^+^ and Zn^2+^) zeolite membranes measured by agarose diffusion assay. (**b**) Antibacterial activity of the zeolites measured by CFU counts on agar plates. (**c**) Fluorescence microscope images of *E. coli* cells on the surface of ion-exchanged zeolites after live/dead staining (syto9 in green, propidium iodide in red, scale presented on the image correspond to 10 µm). The surface is observed using laser reflection (gray levels). (**d**) Quantification of the overall live populations (green) compared to dead populations (red). (**e**) Integration of the data presented in (**d**) showing the live/dead ratio and the total amount of bacteria on the zeolite surfaces.

We used an agarose diffusion assay to assess the ability of the zeolite complexes to inhibit the growth of bacteria through diffusion. The results show that zeolite membrane exchanged with Ag^+^ was capable of inhibiting the growth of *E. coli* bacteria. Distance of the inhibition zones and standard deviations are shown in Figure 7a. The largest inhibition zones were observed for Ag^+^ exchanged zeolite (1.6 mm). There was no inhibition halo around the other zeolite membranes.

#### 2.3.2. Impact on the Colony Forming Activity of a Bacterial Suspension

The impact of the zeolite complexes on the colony forming activity of a bacterial suspension (Figure 7b) was assessed. Support without zeolite coating was used as a negative control. Un-exchanged zeolite (sodium form) shows no antibacterial activity, exhibiting the same Colony Forming Units (CFUs) as the control. No CFUs were observed from the solution incubated with the Ag^+^ zeolite membrane, meaning that all bacteria were killed during the incubation. This demonstrates the strong bactericidal properties of the zeolitic membrane doped with Ag^+^. A significant decrease in colony forming activity was observed for the bacterial solutions incubated with Zn^2+^ exchanged zeolite membrane, suggesting a significant antimicrobial activity for this membrane.

#### 2.3.3. Live and Dead Bacteria Quantification on the Zeolite Surfaces

The viability of bacteria on the zeolite surfaces by fluorescence microscopy was monitored using a syto9/propidium iodide staining method. Cells with a compromised membrane that are considered dead or dying exhibited a red fluorescence whereas cells with an intact membrane will stained green (Figure 7c). On Ag^+^ exchanged membrane surface, almost all bacteria were found dead, comforting our previous results. Interestingly, Zn^2+^ exchanged zeolite membrane showed less bacteria than the un-exchanged zeolite membrane (Na^+^ form) surface, but most cells were alive, suggesting bacteriostatic rather than bactericidal properties (Figure 7d,e).

## 3. Materials and Methods

### 3.1. Materials and Reagents

All chemicals were used as received without further purification: LUDOX AS-40 colloidal silica (40 wt.% in water) was used as Si source, whereas Sodium Aluminate ((50–56 wt.% Al_2_O_3_, 40–45 wt.% Na_2_O,) was used as the Al source zeolite synthesis. Anionic detergent (Alconox^®^) was used to increase the anchoring of zeolite particles on macroporous α-Al_2_O_3_ support. Silver nitrate (AgNO_3_) was used as Ag^+^ source for cation exchange process. All of these reagents were purchased from Sigma-Aldrich (Saint Louis, MO, USA). Zinc chloride (ZnCl_2_, ≥98 wt.%) was purchased from Fluka (Illkirch, France) and used as Zn^2+^ source for cation exchange process. Other chemicals used in this work are sodium hydroxide (NaOH, >99.9 wt.%, Carlo Erba (Val-de-Reuil, France)), and deionized water (~18 mΩ.cm). Macroporous α-Al_2_O_3_ plates 8 × 8 cm^2^ (2 mm thick, 1 µm pore size, 99% porosity) were purchased from FINAL Advanced Materials (Didenheim, France) and cut to 2 × 2 cm^2^ plates. The chemical composition of macroporous α-Al_2_O_3_ support was determined by PANalytical X-ray Fluorescence spectrometer (XRF) (PANalytical, Limeil-Brévannes, France) which is listed in Table 2.

### 3.2. Pretreatment of the Support

Firstly, the anionic detergent (Alconox^®^, 3 g) was dissolved in 400 mL of deionized water heated to 60 °C according to the procedure reported elsewhere [38,39,40]. Subsequently, the surfaces of the macroporous α-Al_2_O_3_ support were cleaned in this Alconox^®^ aqueous solution for 1 h. This step aims to create a higher number of anchoring points for zeolite nuclei on the surface of the alumina during the synthesis step. Finally the plates were removed from the solution, rinsed with distilled water and dried at 70 °C before being cooled down to ambient temperature.

### 3.3. Hydrothermal Synthesis of FAU-Type Zeolite Membranes

In order to prepare the FAU-type membranes, a clear synthesis solution with the molar ratio of 70 Na_2_O:1 Al_2_O_3_:20 SiO_2_:2000 H_2_O [50] was obtained by mixing aluminate solution (S1) and silicate solution (S2) at 70 °C according to an adapted procedure from previous paper [51]. In the first step, sodium hydroxide was dissolved in deionized water at room temperature, then sodium aluminate was added to obtain the solution (S1). Solution (S2) was prepared by mixing LUDOX AS-40 colloidal silica and deionized water at 70 °C. The solution (S2) was added into the solution (S1) under vigorous stirring. After few minutes, a clear, homogeneous solution was obtained. The resulting mixture was stirred overnight at room temperature. The treated or non-treated α-Al_2_O_3_ supports were horizontally placed in a Teflon-lined stainless steel autoclave, and then the synthesis solution was poured into the autoclave and heated for 4 to 24 h at 75 °C. After the hydrothermal growth, the prepared zeolite FAU membrane were rinsed with deionized water and placed in an ultrasonic bath for 5 min to remove the loosely attached crystals and finally dried at 110 °C overnight.

### 3.4. Ion-Exchange

FAU-type zeolite membranes obtained at 75 °C after 24 h of thermal treatment were modified by exchanging the sodium compensating cations present in the parent zeolites with silver (Ag^+^) or zinc (Zn^2+^) cations by a cationic exchange process using silver nitrate (AgNO_3_) or zinc chloride (ZnCl_2_) aqueous solutions (1 M). Typically, FAU-type zeolite membrane was blended with the 1 M cationic aqueous solution that was prepared by mixing AgNO_3_ salt (2.6 g) or ZnCl_2_ salt (2.73 g) with 20 mL of demineralized water. The reaction mixture was then heated at 80 °C for 2 h under stirring. The mass ratio of the reaction mixture is 1 g of zeolite for 20 mL for electrolyte aqueous solution. The pH value of the mixtures was between 6 and 7. After completion of the ion exchange reaction between the Ag^+^, Zn^2+^ and Na^+^, the zeolites membranes were washed 3 times under stirring with cold demineralized water (~200 mL). As silver cation is sensitive to lights and heats, the Ag exchanged FAU membranes were dried in the dark at 80 °C under nitrogen. After each cationic exchange the samples were characterized.

### 3.5. Agarose Diffusion Assay

*E. coli* strain was grown overnight (ON) in Luria Bertani (LB) broth in an incubator shaker at 37 °C (200 rpm). Then, the culture was diluted at 10^8^ bacteria/mL and 100 µL were spread evenly onto LB agar Petri dishes. Lids were left open for 5 min in a laminar flow cabinet to allow for any excess surface moisture to be absorbed into the agar. Zeolite membranes were applied to the surfaces and the plates were inverted and incubated at 37 °C ON to allow for bacterial growth.

### 3.6. Colony Forming Unit Assay

*E. coli* strain was pre-grown ON in LB broth at 37 °C (200 rpm). The bacterial culture was diluted to 0.1 optical density units (OD, measured at λ = 600 nm) and incubate at 37 °C (incubator shaker, 200 rpm) until it reached 0.6 OD. Zeolite samples were inoculated with 3 mL of a 1/100 dilution of the bacterial solution in 35 mm Petri dishes and incubated ON. We performed serial dilutions from the resulting culture and plated 10 μL onto LB agar plates in triplicate. The plates were then incubated at 37 °C for 24 h and CFUs were counted.

### 3.7. Live/Dead Assay

An ON bacterial culture was diluted to 0.1 OD and incubate at 37 °C until it reached 0.6 OD. Zeolite samples were inoculated with 3 mL of a 1/100 dilution of the bacterial solution in 35 mm Petri dishes and incubated ON. We used the LIVE/DEAD *Bac*Light^TM^ Bacterial Viability Kit (Thermofisher, Waltham, MA, USA) to quantity the number and the viability of bacteria on the zeolite surfaces. Briefly, zeolite samples were stained with a PBS solution (phosphate buffered saline, pH 7.4) containing SYTO9 and propidium iodide fluorophores for 15 min followed by a 5 min wash step in PBS alone. A confocal laser scanning microscope (LSM800, Zen software, Zeiss, Marly le roi, France) was used to visualize the stained bacteria. Image analysis was done using ImageJ. Graphs were generated using Prism 7 (GraphPad software, San Diego, CA, USA).

### 3.8. Characterization

X-ray diffraction (XRD) patterns were collected on a PANalytical X’PertPro diffractometer (Limeil-Brévannes, France) using CuKα radiation (λ = 1.5418 Å) and θ-θ mounting at room temperature under ambient pressure. The patterns were registered in 2θ range from 5° to 50° with a scanning step of 0.013° 2θ and a time per step of 200 s. The Si/Al molar ratio was determined from the refinement of the unit cell parameters of the non-calcined films. From these parameters, the Si/Al framework ratios of the zeolite layer were deduced using the Breck and Flanigen [37]. The morphology, homogeneity and thickness of different zeolite films were determined with Scanning Electron Microscopy (SEM) using a Philips XL-30 FEG microscope (FEI-Thermo Fisher Scientific, Eindhoven, Netherlands). Energy dispersive X-ray (EDX) analyses were a JEOL JSM-7900F microscope (Croissy sur Seine, France) equipped with BRUKER QUANTAX EDX spectrometer (Synergie4, Evry, France). In order to investigate the cations distribution in our materials, element maps were collected at 6 kV (for AgX zeolites) or 5 kV (for ZnX zeolites) accelerating voltage.

The porosities of the uncoated and coated alumina plate were analyzed by mercury intrusion porosimetry using an Autopore IV porosimeter (Micromeritics, Merignac, France). This device allows working at pressures between 2.6 × 10^−6^ and 400 MPa. Before the measurements, the alumina plates were degassed at 300 °C under vacuum (100 Pa) for approximately 12 h. About 300–800 mg of degassed alumina plate (coated or not) were introduced into the penetrometer, which was then placed in the low-pressure chamber (2.6 × 10^−6^–0.2 MPa). During this first step, the cell was evacuated and filled with mercury. The penetrometer containing broken pieces of uncoated or coated alumina plates and mercury was then placed in the high pressure chamber (0.2–400 MPa). During this second step, pressure was applied to force the diffusion of mercury into the porous sample. As the intrusion occurs, the level of mercury in the stem varies. The Washburn equation [51] was used to process the data: P × D = 4σcosθ, where P is the applied pressure (Pa), D is the pore diameter (m), σ is the interfacial tension (N·m^−1^), and θ the contact angle (°) (for mercury σ = 485 mN·m^−1^ and θ = 130°). Nitrogen adsorption-desorption isotherms were performed at −196 °C using ASAP 2420 apparatus (Micromeritics, Merignac, France). Prior to each manometric experiment, the zeolitic samples were outgassed under vacuum for 1 h at 90 °C then 15 h at 300 °C to eliminate physisorbed water and VOC molecules. The microporous volume (V_micro_) was calculated by t-plot method.

## 4. Conclusions

In this work, a FAU-type zeolite layer was synthesized on macroporous alumina plates. After gentle cleaning to make the plate surfaces rougher, a 24 h in situ hydrothermal treatment proved to be the optimal condition for obtaining a very good quality membrane. A dense and continuous layer of FAU-type zeolite with a thickness of 2.5 μm was formed directly on the surface of the alumina support, without any crack. An Si/Al molar ratio of 1.2 and the crystal morphology of the FAU-type zeolite were determined. The accessibility of the porosity of the formed film was verified by N_2_ physisorption analysis. A simple and fast method has therefore been developed to synthesize very good quality FAU-type zeolite membranes. Zeolites membranes exchanged with Ag^+^ showed an agar-diffusive bactericidal activity against gram negative *E. coli* bacteria. Zn^2+^ exchanged zeolites presented a bacteriostatic activity that is less diffusive in agar. As expected, non-exchanged zeolite membrane (in its Na^+^ form) have no effect on bacterial activity.

## Figures and Tables

**Table 1:** The results of N_2_ physisorption of the zeolite layers obtained on treated macroporous α-Al_2_O_3_ supports, after 24 h of synthesis at the heating temperature at 75 °C.

Zeolite-Type	Adsorbed Volume ^a^ (cm^3^/g_sample_ STP)	V_micro_^b^ Zeolite Layer (cm^3^/g_sample_)	Zeolite Weight on Support ^c^ (mg)
FAU	1.04	1.6 × 10^−3^	~22.9

^a^ Deduced from N_2_ adsorption isotherm at relative pressure of 0.1. ^b^ Microporous volume of the zeolite layer determined; value determined by the t-plot method. ^c^ Determined from Equation (2).

**Table 2:** Chemical Composition of macroporous α-Al_2_O_3_ plate.

Chemical Component	Mass Fraction (%)
Al_2_O_3_	98.63
ZrO_2_	0.64
SiO_2_	0.30
CaO	0.12
Fe_2_O_3_	0.09
K_2_O	0.06
Na_2_O	0.05
Others	<0.01
